# An App-Based Intervention for Adolescents Exposed to Cyberbullying in Norway: Protocol for a Randomized Controlled Trial

**DOI:** 10.2196/31789

**Published:** 2021-11-08

**Authors:** Sabine Kaiser, Monica Martinussen, Frode Adolfsen, Kyrre Breivik, Henriette Kyrrestad

**Affiliations:** 1 Regional Centre for Child and Youth Mental Health and Child Welfare - North UiT The Arctic University of Norway Tromsø Norway; 2 Regional Centre for Child and Youth Mental Health and Child Welfare - West, NORCE, Norwegian Research Center AS Bergen Norway

**Keywords:** cyberbullying, intervention, mobile app, adolescents, NettOpp, mental health, adolescents, health care

## Abstract

**Background:**

Adolescents exposed to negative online events are at high risk to develop mental health problems. Little is known about what is effective for treatment in this group. NettOpp is a new mobile app for adolescents who have been exposed to cyberbullying or negative online experiences in Norway.

**Objective:**

The aim of this paper is to provide a description of the content of the intervention and about a randomized controlled trial that will be conducted to examine the effectiveness of NettOpp. This protocol is written in accordance with the Spirit 2013 Checklist.

**Methods:**

An effectiveness study with a follow-up examination after 3 months will be conducted to evaluate the mobile app. Adolescents will be recruited through schools and will be randomly assigned to the intervention (NettOpp) group and a waiting-list control group. The adolescents (aged 11 to 16 years) will respond to self-report questionnaires on the internet. Primary outcomes will be changes in mental health assessed with the Strengths and Difficulties Questionnaire, the WHO-Five Well-being Index, and the Child and Adolescent Trauma Screen.

**Results:**

Recruitment will start in January 2022. The results from this study will be available in 2023.

**Conclusions:**

There are few published evaluation studies on app-based interventions. This project and its publications will contribute new knowledge to the field.

**Trial Registration:**

ClinicalTrials.gov NCT04176666; https://clinicaltrials.gov/ct2/show/NCT04176666

**International Registered Report Identifier (IRRID):**

PRR1-10.2196/31789

## Introduction

### Background

The technological revolution has led to most young people being able to now use the internet and mobile phones daily to communicate with others, socialize, entertain themselves, and find information. This development goes hand in hand with many new challenges and possibilities also when it comes to aversive behavior among children and youth. On the one hand, cyberbullying and other negative online experiences affect many children and youth. On the other hand, this technological development opens up for new innovative technologies to help and support young people exposed to such behavior.

Cyberbullying can be defined as an “aggressive, intentional act carried out by a group or individual, *using electronic forms of contact*, repeatedly and over time against a victim who cannot easily defend him or herself” [1]. Electronic forms can be the internet and other digital technologies including mobile phones that are used to, for example, call, write emails, instant, and text messages, chats, blogs, or web posts to say mean things, insult, threaten, or make fun of somebody, to spread rumors, lies, embarrassing information, or pictures [2]. Cyberbullying can take on many different forms, from passively ignoring or excluding somebody from a group to more active actions such as sending or posting cruel or embarrassing messages about someone [2,3].

Cyberbullying prevalence rates among 12-to-18-year-old individuals vary from 5% to 74% with a median of 23%, as reported in a review by Hamm et al [4]. Some of this variation can probably be explained by age group and country differences in the prevalence of cyberbullying. Nonetheless, we believe it is likely that this huge variation is also due to the use of different definitions, perceptions, and interpretation for cyberbullying across studies; that is, does cyberbullying have to occur repeatedly or is one occurrence enough [3-6] and what are the different types of scales used to measure the phenomenon? The annual conducted Norwegian school survey among 10-to-18-year-old students reported a cyberbullying rate of 2% in 2017 with a peak (2.6%) in junior high school [7]. This is the proportion of adolescents who report that they have been cyberbullied 2-3 times a month or more. The proportion increases to 10% if adolescents who have experienced one negative online event are included.

The consequences of bullying victimization in adolescence are serious. Meta-analyses have found that bullying, both traditionally and on the internet, is related to mental health problems such as depression, anxiety, and poor general health [8,9]. Furthermore, there is an association between bullying victimization and psychosomatic health complaints such as stomach ache, sleeping difficulties, and headache and social functioning including social isolation, loneliness, and low self-esteem [2,8,10].

Given the seriousness of bullying or cyberbullying victimization, interventions that aim at preventing cyberbullying and helping and supporting those exposed to cyberbullying are important. A recently published meta-analysis found that intervention and prevention programs for cyberbullying can reduce cyberbullying victimization [11]. Furthermore, some traditional antibullying programs have also proven to have an effect on cyberbullying [12,13].

A review found that there are more preventive antibullying programs compared to interventions for adolescents who have been exposed to cyberbullying [10]. However, such studies suggest that cognitive measures appear to be effective [10]. A systematic review of digital bullying from the Norwegian Institute of Public Health did not identify any available interventions in Norway, except for 2 anticyberbullying campaigns [14]. Those campaigns were neither theoretically grounded nor evaluated. The report encourages using technology and being innovative when developing measures to prevent cyberbullying [14] as adolescents spend a lot of their time on the internet and with their mobile phones [15]. Furthermore, many adolescents find it difficult to tell their parents or other adults about their experiences of being bullied or cyberbullied [16,17]. Therefore, a mobile app may be a useful resource as they are always accessible, easy to use, and they offer anonymity.

Several health-promoting apps aimed at youth have been developed. Many of them aim to promote health by monitoring or motivating the user to adopt healthier diets or increase their physical activity [18]. Other apps are more supportive in which the purpose is to learn how to cope with, for example, chronic diseases such as diabetes, asthma, or cancer [19]. Overall, two reviews concluded that apps may have the potential to be feasible health interventions for young people, but that more studies are needed to assess their effectiveness [19,20]. In addition to this, Shieh (2016) has found 9 anticyberbullying apps with different focus [21]. However, none of these apps have been empirically evaluated or adapted to Norwegian conditions, indicating the need for the development and evaluation of an app against cyberbullying in Norway.

### The Cyberbullying Coping Intervention

**Figure 1:**
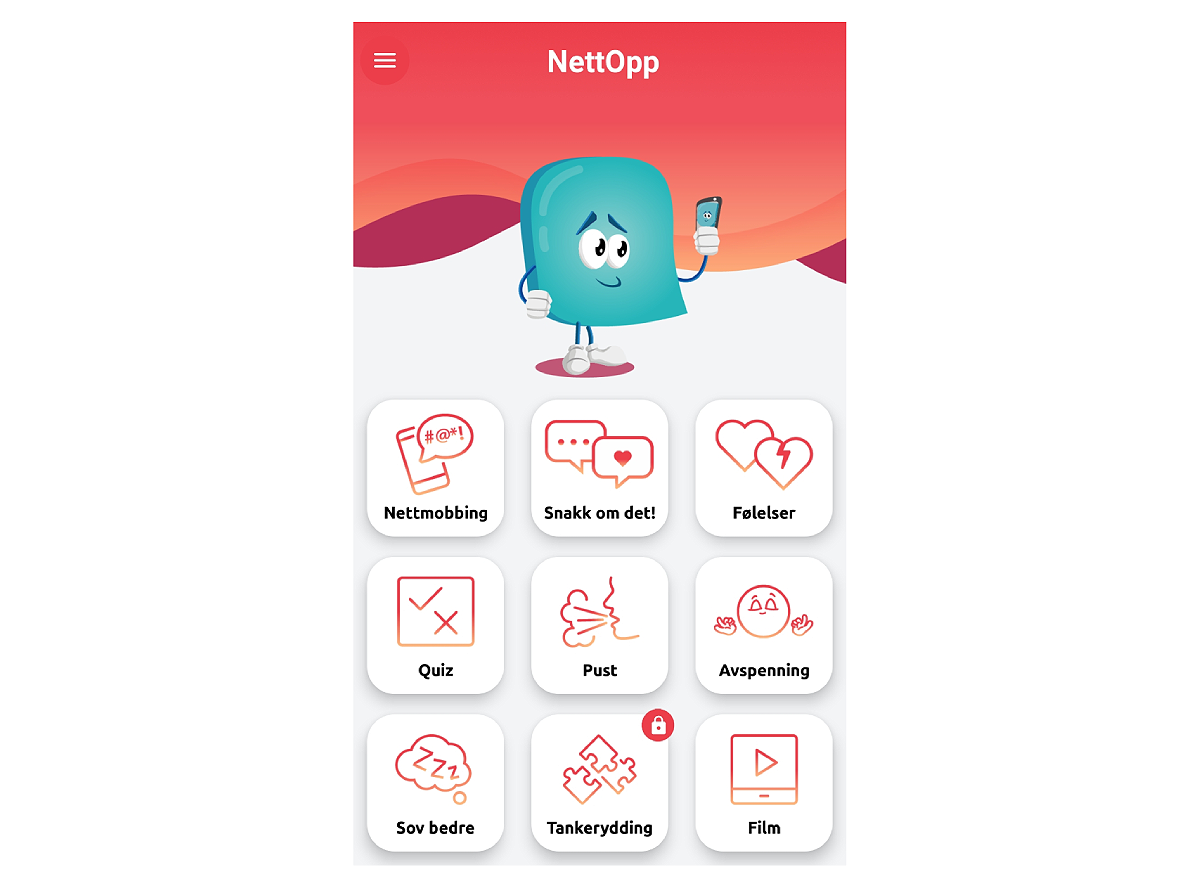
NettOpp start page.

NettOpp is a mobile app for adolescents who have been exposed to cyberbullying or other negative online experiences in Norway (Figure 1). NettOpp directly translated means “exactly” in English but “Nett” refers also to the internet and “Opp” comes from “Opplysning,” which means information or enlightenment. The target group of the cyberbullying coping intervention was adolescents in elementary school and junior high school, between 11 to 16 years of age. According to Jacobs et al [22] (2014), a cyberbullying coping intervention should do more than just increase awareness about internet threats. Ideally, an intervention should reduce the risks for getting victimized, combat cyberbullying once it occurred, and buffer the negative impact of the event [23].

The primary aim of this intervention is (1) to reduce mental health problems related to cyberbullying; the secondary aims are to (2) increase adolescents coping skills with cyberbullying, (3) increase knowledge about cyberbullying, (4) increase the help-seeking behavior of the adolescents, (5) increase their self-esteem, (6) increase their sleeping quality, and finally, (7) reduce cyberbullying. Below a description of why it is important to focus on the aforementioned aims.

A previously conducted user survey (N=15) showed that most users found NettOpp easy to use, appealing, and would recommend the mobile app to a friend. A total of 14 out of 15 adolescents agreed that NettOpp would probably increase knowledge about cyberbullying and 5 out of 15 believed that NettOpp would reduce cyberbullying [24].

### Reduce Mental Health Problems

Adolescents exposed to cyberbullying are at high risk for developing mental health problems. The intensity and duration of the cyberbullying may determine how serious the consequences are [25]. Giving the adolescents advice on how to cope with cyberbullying might have a buffering impact and can reduce mental health problems. A study found that, for example, help-seeking behavior buffered the negative impact of cyber victimization on depressive symptoms [26] and internalizing problems [27]. In addition, learning how to deal with a cyberbullying event may shorten the cyberbullying episode and thereby prevent serious consequences that could affect the health of the adolescents.

### Increase Adolescents’ Coping Skills With Cyberbullying

How an adolescent copes with a cyberbullying event can determine whether he/she experiences long-term consequences [22]. A study found that among the most helpful coping strategies to stop cyberbullying were technical strategies (eg, blocking or deleting the person or profile), help-seeking behavior, and behavioral avoidance (eg, stopping visiting the webpages where the event happened) [28].

Furthermore, cyberbullying is emotionally distressing and young people exposed to it can feel upset, hurt, embarrassed, helpless, isolated, and scared for their safety [29]. Teaching adolescents about normal emotional reactions to a cyberbullying event and how to cope with the feelings could be emotionally helpful. Healthy coping strategies (eg, help-seeking and talking about the problem or using relaxation techniques, listening to music, or thinking positive thoughts) could replace unhealthy coping strategies (eg, social withdrawal, self-harm, aggression, or skipping school), and buffer the negative impact of the cyberbullying event [23]. A study found that among the most emotionally helpful coping strategies for cyberbullying victims were support-seeking, technical strategies, behavioral avoidance, and reframing the situation (eg, “whoever is doing this to me is not worth my time”) [28].

### Increase Knowledge About Cyberbullying

Increasing knowledge about what cyberbullying is, what consequences are associated with it, rights and laws, security advice, and what can be done in the case of a cyberbullying event is an important part of the psychoeducation adolescents should receive [25,30]. It will increase awareness of the problem, its seriousness, and help the adolescent to act safer on the internet and thus reduce the risk of being victimized in the future. Providing information on how to deal with an event might help the adolescent to combat cyberbullying once it occurred.

### Increase Help-Seeking Behavior Among Adolescents

Some studies that examined help-seeking behavior among individuals exposed to traditional bullying have found that telling an adult made things worse for some adolescents [27,31,32]. Regarding cyberbullying, Price and Dalgleish [33] found that the majority (approximately 60%-70%) of adolescents who sought help found this helpful to some degree. For the remaining individuals, help-seeking did not change the situation. Price and Dalgleish [33] concluded, “a critical response to effectively addressing cyberbullying relies on both increasing the help-seeking behaviour of victimized young people and improving the efficacy of those they speak to.” Seeking support was also found to be emotionally helpful for the majority of victims of cyberbullying and helped to stop cyberbullying for some adolescents in another study [28]. However, some adolescents do not have a trusted adult around, or they are too ashamed or afraid to tell somebody they know and may prefer to contact someone anonymously [23]. Encouraging adolescents to seek help, provide information about whom to contact (eg, also about online resources and helplines), how to seek help, and what should happen when adolescents have sought help at school are important [22].

### Increase the Self-esteem of the Adolescents

Low self-esteem in adolescence has been found to predict negative consequences such as poor physical and mental health during adulthood [34]. Studies have found a negative relationship between cyberbullying victimization and self-esteem [35-37]. Interventions that aim to increase self-esteem are therefore important. Most of the studies that aim at increasing self-esteem use physical exercise as the intervention [38].

### Increase the Sleeping Quality

Insufficient sleep among adolescents is related to physiological and mental health risks such as cardiometabolic dysfunction, poor academic performance, or mood disturbances such as increased suicidal ideation [39], and it was found to be a precursor to depression [40]. A meta-analysis found that peer victimization like bullying among children and adolescents was related to sleeping problems [41]. Healthy lifestyles including longer sleep duration, on the other hand, was associated with less suicidal ideation among individuals exposed to cyberbullying [42]. In general, a review and meta-analysis found that cognitive-behavioral sleep interventions for adolescents are effective in improving sleeping quality [43].

### Reduce Cyberbullying

Cyberbullying rates can be reduced by increasing the knowledge and skills about how to handle cyberbullying [25]. The knowledge adolescents will acquire about what cyberbullying is, what consequences are associated with it, and rights and laws may lead to increased awareness of the problem. By teaching the adolescents how to reduce risks of being victimized and how to better cope with a cyberbullying event might help to stop cyberbullying and prevent new occurrences and thus reduce cyberbullying rates.

### The Mobile App

The app will consist of 2 modules. Module 1 will be psychoeducational including information about cyberbullying, its consequences, rights and laws, practical and technical advice about how to cope with a cyberbullying event (eg, blocking or deleting a person). This will hopefully increase adolescents’ knowledge about cyberbullying. The app will also provide tips about what the adolescents can do to reduce new occurrences of cyberbullying events (eg, “Don’t add ‘Friends’ that you don’t know who is,” “Be critical of which images you share with others,” and “Check your privacy settings on social media”). In addition, the adolescents will learn about normal emotional reactions when exposed to cyberbullying (eg, fear, helplessness, shame, and sadness) and how to cope with them. This will contribute to increased coping skills with cyberbullying. Further, the psychoeducational information focuses on motivating adolescents to seek help from a trusted adult. Adolescents are advised to contact a trusted adult who they think can help in case of cyberbullying or a negative online experience. The app further informs about how one can talk about difficult things to someone else and about what should happen once the adolescent has told an adult. In addition, there is information about available professional online resources including chats and helplines the adolescents can contact in case they do not know with whom to talk or prefer to stay anonymous. This might increase help-seeking behavior among adolescents.

Module 2 will be a resource module that provides exercises and techniques on how to cope with emotional distress related to cyberbullying. These exercises include relaxation techniques (breathing and guided meditation exercises) that aim at increasing the adolescents’ coping skills with the cyberbullying event. The exercises also include sleep hygiene–related advice and exercises to increase the sleep quality of adolescents that have, for example, difficulties to fall asleep because of worries. The adolescents will be encouraged to create a bedroom where they feel safe, enjoy themselves, and relax. They will also be encouraged to relate to their concerns and quarry thoughts during daytime.

The app also includes an exercise based on cognitive behavioral therapy, which aims at helping the adolescents to reframe the situation [44]. The aim is to make the adolescents aware of the connection between thoughts and feelings, and at increasing awareness about negative thoughts the adolescents might have because of the cyberbullying event and replacing these thoughts with alternative thoughts. The adolescents will be guided through this exercise by giving them the opportunity to choose between different statements. First, they can choose a statement that fits best to their situation (eg, “Someone has posted a picture or video of me that I don't like”). Then, they can identify the negative thoughts (eg, “I'm thick/ugly/stupid” and “Everyone is going to think I look stupid”) and rate how strongly they believe in this thought on a scale from 0 to 10. In a third step, they can identify the negative feelings they are experiencing (eg, “I feel sad and tired” and “I'm ashamed”) and rate how strongly they experience this feeling on a scale from 0 to 10. Thereafter, they will be shown a list of alternative thoughts. They will be instructed to pick out alternative thoughts or advice on what they could do, what they can say to themselves, and what they would have said to a friend who was in a similar situation. They can select up to 3 statements (eg, “It’s not my fault that I’m being exposed to this,” “I can ask for help [e.g., from parents, teachers, health nurses],” and “There are many people that like and care about me”). In a last step, the adolescents can rate how strong the original, negative thought and the feelings are and rate once more if they feel better, the same, or worse. The overall aim of this and the other exercises is to increase coping skills with a cyberbullying event and thus to prevent the development of mental health problems.

Furthermore, every second day, the adolescents will receive a push-message in the app, which will either say something nice (eg, “Do something nice for yourself”) or motivate the adolescents to do an exercise (eg, “Relax and do a breathing exercise”).

Information will be displayed in the app through text, sound recordings, and short movies, to keep the adolescents engaged with the app. In addition, rights and laws associated with cyberbullying will be communicated to the adolescents through quizzes.

### User Involvement

The users, in our case Norwegian elementary and junior high school students, have been involved in the project from the development of the intervention to its evaluation. The app was developed in collaboration with the users in terms of what the intervention should contain, but also in terms of functionality and format of the app. The 8 adolescents in the user group were involved through workshops. A larger reference group consisted of teachers, school health nurses, nurses from the health care station for adolescents, community psychologists, and the local antibullying professional.

The aim of the current paper is to provide a description of the content of the intervention and the randomized controlled trial that will be conducted to examine the effectiveness of NettOpp.

## Methods

### Eligibility Criteria and Setting of the Effectiveness Study

Adolescents from the 6th to 10th grade (11-16 years old) are eligible for participation in the effectiveness study. Adolescents will be recruited through schools in Norway. To recruit enough participants, the schools need to be big enough; that is, they should have at least 20 students in each class.

### Inclusion and Exclusion Criteria

Adolescents between the ages of 11 and 16 years, whose guardians have given consent and who agree to take part in the study, will be included. To use the app, adolescents need a smartphone and be able to read and understand Norwegian. Furthermore, android users must provide a Gmail address, and iPhone users must first download the free app TestFlight to be able to download a test version of NettOpp. Adolescents who may not benefit from an app-based intervention because of, for example, severe developmental or cognitive challenges will be excluded from the study.

### Intervention

NettOpp is a self-help tool that aims at supporting adolescents who have been exposed to cyberbullying or a negative online event. Adolescents can install the app on their mobile phone and use it as much as they want and whenever they want. The intervention focuses on psychoeducation, on motivating the adolescents to seek help from a trusted adult, and on strategies to better cope with stress related to cyberbullying or negative online experiences.

### Control

The waiting-list control group will receive access to the app after study completion; that is, when the follow-up assessment is conducted after approximately 3 months.

### Randomization

The effectiveness study will be conducted as a randomized controlled trial with an intervention group and a waiting-list control group. Randomization will be conducted after baseline measures have been collected at the school level by a statistician. A random number between 0 and 1 will be generated using SPSS and assigned to each school. Half of the schools with the highest value on the random variable will be assigned to the intervention group and half of the schools with the lowest value will be assigned to the waiting-list control group.

### Blinding

Adolescents are randomized to the intervention or waiting-list control group and are blinded to the allocation prior to the baseline assessment, and their schools will also not receive information about their allocation. The information letters include information about study content and purpose, but guardians and students were both blinded to the allocation to the intervention or waiting-list control group prior to offering their consent or before the baseline assessment.

### Outcomes

Data will be collected at baseline (T_1_, preintervention) and after approximately 2 weeks of the intervention (T_2_, postintervention) through self-report measures that the adolescents fill in using Nettskjema, a secure online tool to conduct surveys [45]. A follow-up evaluation (T_3_) will be conducted after approximately 3 months to examine if the effects were stable over time.

### Primary Outcomes

Mental health will be assessed with the Strengths and Difficulties Questionnaire (SDQ) [46], the WHO-Five Well-being Index (WHO-5) [47], and the Child and Adolescent Trauma Screen (CATS) [48]. The null hypothesis of this study is that there will not be significant differences in changes in mental health scores between the waiting-list control and the intervention group.

### Secondary Outcomes

How the adolescents cope with cyberbullying will be measured with the Cyberbullying Coping Questionnaire [49]. Help-seeking behavior will be assessed with 3 questions; for example, “Have you told someone about your experiences so they can help you?” Health problems will be assessed using 7 items asking the respondent how often he/she has, for example, experienced headaches. Self-esteem will be measured with the Norwegian Version of the Self-liking and Competence Scale [50,51]. Sleeping quality will be measured with 6 questions (eg, “At what time do you usually go to bed?”) from the Bergen Child Study [52]. Cyberbullying and bullying experiences will be assessed using 4 questions based on the Olweus questionnaire [5].

### Power Calculations

Power calculations were conducted using the software PASS 16 [53]. Using multilevel analysis, it will require a total sample of 400 participants (200 in each group: 20 schools with 20 students per group) to detect an effect size of at least Cohen *d*=0.30, when the expected interclass correlation at school level is 0.01, with a power of 0.79, and a significance level of .05.

### Data Management

Data collection, data cleaning, and statistical analyses will be performed by members of the research team. The statistical analyses will be conducted on anonymized data and only members of the research team will have access to the data. Data will be stored on a secure server.

### Planned Statistical Analysis

A linear mixed model will be used for analyzing the outcomes in the effectiveness study. Missing data will be handled using multiple imputation.

### Ethics Approval and Consent to Participate

The effectiveness study is approved by the regional Research Ethics Committee (reference number 161212). The studies are approved by the Norwegian Centre for Research Data (NSD) (reference number 545417). Since the study participants are between 11 to 16 years old, study participation requires consent from an authorized guardian. The consent form and information letter to the guardians and study participants are approved by the regional Research Ethics Committee and by the NSD. Changes to the project, which may impact study participants will be reported to the regional Research Ethics Committee and to the NSD.

## Results

The study has been approved by the regional Research Ethics Committee and by the NSD. The mobile app NettOpp has been developed, and enrollment for the study will begin in January 2022. The results of the study will be published in 2023.

## Discussion

### Expected Outcomes

Adolescents who have been exposed to cyberbullying are vulnerable to mental health problems and other harmful effects of the stressful events. In addition to the negative effects that cyberbullying can have for those who have been exposed to it, there may be a considerable financial burden to the society, which is associated with the consequences of mental health problems in adolescence and over the lifespan [8,54,55]. The threshold for telling and seeking help from a trusted adult might be too high and as such, there is a need for a low-threshold intervention against cyberbullying for adolescents.

NettOpp is a mobile app for adolescents who have been exposed to cyberbullying or a negative online experience in Norway. Its evaluation will contribute unique knowledge to the field as there are very few interventions targeting adolescents who have been exposed to cyberbullying. The aim of this paper is to provide a description of the content of the intervention and about its evaluation. The results of the evaluation will be presented in other studies. If the intervention is found to be effective, it will be free of charge and available to all adolescents in Norway.

### Challenges

The intervention and its evaluation have several limitations. First, the intervention may be too comprehensive, aim at too many areas (ie, knowledge, coping, self-esteem, sleep, mental health, and reduce cyberbullying rates), and may partly contain too much information. In particular, the exercise where the adolescent learns about unhelpful or inappropriate thoughts and reframing the situation is long and is based on written information. This might not be appealing to adolescents and that they will therefore not use it. However, it is difficult to redesign this exercise to make it more attractive. Furthermore, we find that the exercise is too important to exclude it from the intervention. The questionnaire to evaluate the intervention is long and may lead to dropping out, response fatigue, or saying no or yes [22]. However, it is necessary to assess these measures to evaluate the effectiveness of the intervention. Both working with the intervention and filling in the questionnaire can be potentially distressing for the adolescents as they are confronted with the seriousness of the cyberbullying event and their thoughts, feelings, and consequences of the event. Therefore, we inform the adolescents about possible contact persons such as school nurses and provide a number to a helpline in the information letter and in the online questionnaire. However, in general, we expect the benefits of the intervention to exceed its disadvantages.

## Abbreviations

CATS: Child and Adolescent Trauma Screen
NSD: Norwegian Centre for Research Data
SDQ: Strengths and Difficulties Questionnaire
UiT: The Arctic University of Norway
WHO-5: WHO-Five Well-being Index
